# Zār Spirit Possession in Iran and African Countries: Group Distress, Culture-Bound Syndrome or Cultural Concept of Distress?

**Published:** 2015-09

**Authors:** Fahimeh Mianji, Yousef Semnani

**Affiliations:** 1 Division of Social & Transcultural Psychiatry, McGill University, Montreal, Canada; 2 Department of Psychiatry, ImamHossein Hospital, Shahid Beheshti University of Medical Sciences, Tehran, Iran

**Keywords:** *Zār*, *Spirit Possession*, *Culture-Bound Syndrome*, *Cultural Concepts of Distress*, *Mental Illness*, *Globalization*, * African Countries*, *Iran*

Zār is the term used to describe a form of spirit possession common in northern African, eastern African, and some Middle-Eastern societies. Although these regions share some cultural similarities arising from their history of slavery, in these places, zār varies in prevalence, clinical characteristics, and social context. Based on a selective review of the literature, this paper looks at the place of zār spirit possession in both DSM-IV and DSM-V; it also examines how zār is manifested in Iran and in African countries including Sudan, Ethiopia, and Egypt; and it aims to provide practical information to mental health clinicians so that they can better understand how this cultural concept is practiced by Iranians and Middle Eastern and African immigrants living near the Persian Gulf coast.

Zār refers to a type of spirit, to the illness caused by those spirits who possess humans, and to the rituals needed to pacify those spirits (1). The zār cult is found throughout northern and eastern African countries such as Sudan (2-4), Egypt (5-8), Ethiopia (9-11), and Somalia, where it is called sar (12, 13), as well as some Middle-Eastern countries such as Kuwait (14, 15), Israel (16) and southern Iran (17-20). In these cultures, spirit possession is associated with dissociative episodes such as sudden changes in consciousness or identity that may include periods of shouting, banging of the head against the wall, laughing, singing, or crying. Possessed people may become apathetic or withdrawn, or may not be able to accomplish their usual responsibilities (21).

In the Diagnostic and Statistical Manual of Mental Disorders (DSM-IV-TR), zār is addressed as a culture-bound syndrome. DSM-IV contains symptomatic descriptions of 25 culture-bound syndromes. According to the Glossary of Culture-Bound Syndromes in appendix I of DSM-IV (p. 844), “[T]he term culture-bound syndrome denotes recurrent, locality-specific patterns of aberrant behavior and troubling experience that may or may not be linked to a particular DSM-IV diagnostic category. Many of these patterns are indigenously considered to be ‘illnesses’ or at least afflictions and most [of them] have local names. . . . [C]ulture-bound syndromes are generally limited to specific societies or culture areas and are localized, folk, diagnostic categories that frame coherent meanings for certain repetitive, patterned, and troubling sets of experiences and observations.” Although zār is not considered pathological in local cultures, depending on its severity, it can be perceived outside of the cults in which it develops as a symptom of more serious mental health problems (22), such as psychosis and/or dissociative identity disorder. 

Similar to DSM-IV, in DSM-V, there are comments on specific culturally shaped syndromes—those few disorders around the world that are truly “bound” to one culture—but there are also discussions about the cultural concepts of illness and a deconstructed notion of culture-bound syndromes, which many believe were a legacy of colonial psychiatry. Based on the most recent change in DSM-V, culture-bound syndromes are actually better understood as some forms of Cultural Concepts of Distress such as cultural syndromes, cultural idioms of distress, and/or cultural explanations. According to DSM-V, “Cultural Concepts of Distress refers to ways that cultural groups experience, understand and communicate suffering, behavioral problems, or troubling thoughts and emotions” (23). This is one of the subjects that changed in DSM-V: rather than the long glossary of different syndromes, including zār, offered by DSM-IV, there are examples providing more details that aim to clarify the concepts of culturally shaped or influenced symptom clusters (syndromes), of common modes of expressing distress that may not involve specific symptoms (idiom), and of explicit causal models, attributions, or explanations (explanatory model), with the acknowledgment that these concepts often overlap (24).

Although in DSM-V the zār is not listed among the examples of the cultural concept of distress, it is still crucial to be aware that in the places where the zār ritual is practiced, the patients’ clinical presentations may be influenced by the cultural concepts and modes of expression explained in DSM-V. People in the cults where zār is believed and practiced use it when they face difficult circumstances such as marital conflicts, gender and social inequalities or in response to social and cultural changes (1, 15). Believing in zār illness and following the zār ritual as a therapy can therefore act as a method of coping with disruptive physiological, psychological, social, or supernatural conditions (16). Although the best known spirit possession within Islam is the zār in northern and eastern Africa, possession cults are common and not confined to Muslims in Africa. Contrary to the common belief, zār is not just an Islamic form of spirit possession, and the spirit possession is not always a positive experience (1, 25). Boddy (1989) has shown that zār is practiced in Christian Ethiopia (which is different from what is practiced in Sudan). Also, Hamer found a similar cult in the south of that country called shatana (devil) (26); and Edelstain studied zār among Jewish Ethiopians in Israel (16).

Similarity in the names of this spirit possession suggests that many of the cults are historically related although evidence has not directly addressed this claim. For example, in Chad, there is the liban sheitan (27, 28); among the Digo of the south Kenya coast, there is shaitani (29, 30); and among Segeju Swahili speakers in Tanzania, there is shetani (31). Moreover, the form of Masabe spirit possession among the Tonga of Zambia (32, 33) resembles the Sudanese zār; the bori in Nigeria and North Africa; trumba and patros in Mayotte, Comoro Islands; saka or pepo among the Wataita of Kenya; takuka among the Ndembu of Zambia; holey (specifically, hauka) among the Songhay; and jnun curing activities, Iamadsha in Morocco (1, 28). Sudanese and West African pilgrims in and en route to Mecca have been seen to participate in each other’s spirit rituals (following the Ottoman conquest in 1820). Moreover, in several cults, possessive spirits and zār are associated with “winds”: pepo in Swahili, iska in Hausa, rih/rowhan in Arabic (1, 34 and 35). Similarly, in southern coastal regions of Iran near the Persian Gulf such as Bandar-e-Abbās, Bandar-e Lengeh and Qeshm Islan, zār is known as the harmful “wind” (bād or Hava) associated with spirit possession beliefs. It is believed that these winds can be either fearsome or peaceful, Muslim or infidel. In these regions, zār is the infidel winds which are considered harmful. Among the local people, there are various winds known zārs, most of which cause disease, discomfort, and mental illness in their victims. Some of these zārs are called Maturi, Šayḵ Šangar, Dingemāru, Omagāre, Bumaryom, Pepe, Bābur, Bibi, and Namrud (19, 20).

Considering the globalization and the migration of a large number of people from rural areas and small cities to larger cities, as well as immigration from low- and middle-income countries to high-income countries, it is important that mental health clinicians familiarize themselves with zār, this formerly-called culture-bound syndrome, and its historical roots in different contexts. Based on a selective review of cultural psychiatry and medical anthropology literature, this paper aims to provide clinicians and researchers with a sociocultural and historical understanding of zār spirit possession that will help them identify this condition in clinical settings in order to avoid misinterpreting it as a psychosis and/or dissociative identity disorder and to increase intervention efficiency.

***Forms and Variations of Zārs in African Countries***

*Ethiopia:*

According to a well-known Ethiopian story, Adam and Eve had thirty children and to protect the most beautiful ones from the Divine envy, Eve tried to hide fifteen of them in the Garden of Eden, out of God’s sight. God, who was all-seeing, got angry with Eve and declared that those children would remain invisible for eternity. The fifteen invisible children became the ancestors of resentful and unpredictable spirits like zārs, and the other fifteen became the ancestors of humanity. In Ethiopia, if a zār chooses a person, then that person would be a member of the zār cult. The zār’s intention almost always would be identified by the physiological symptoms that have not effectively responded to the existing medical options. The most common symptoms include headaches, lethargy, or infertility, which different types of healers, including biomedical doctors, could not treat (9, 16). Researching on group psychotherapy in an Ethiopian possession cult, Allan Young describes that the Amhara are Christian minorities who mainly live in the central and northern provinces of Ethiopia such as Shoa, Gojam, Wollo, and Begemder. Traditionally, Amhara men have such occupations as farmers, clergy, civil servants, soldiers, and petty traders. Young also emphasizes that the spirit possession cult is not a unitary phenomenon throughout this area and that there are important ritual and cosmological differences from place to place. In most African areas where zār is prevalent, women are the majority of the sufferers, whereas in Ethiopia, a greater proportion of men are afflicted with zār. 

Although spirit-possession-related illnesses are treated as an ancillary concern by most cult members, in the Amhara, zār-related illnesses are considered important in terms of the relations between cult spirits and cult members both during and after initiation. In fact, participating in illness episodes provides members with the adaptive strategies that the cult offers (9).

Egypt: In a study on the spirit possession cult in Nubia, Egypt, Kennedy (1967) provided a clear picture of the Nubian zār cult mentioning several methods that the Nubians use to deal with their health disturbances. Among those methods, zār is known as the most powerful therapeutic practice; it is effective for several types of illnesses and is specialized for alleviating states of hysteria and anxiety. According to the Nubian disease theory, illnesses are caused by the evil eyes of envy, the breaking of taboos, and sorcery, which all are associated with spirits; mental illnesses are mainly caused by spirits or jinn. Nubians believe that the spirits or jinn occupy rooms and houses when no one is at home. They will then enter the person’s body—specifically angry or aggressive persons, who are more attractive to the jinn. However, the spirits or jinn, rather than the possessed person, would be blamed for the erratic behaviour (6, 36). The Nubian zār ritual deals with the demonic powers of bad spirits such as sheytan, jinn, or zār. Similar to the Ethiopians, Egyptians use the zār ritual only when other curing methods have not succeeded. It means that the demons have succeeded. The ceremony aims to persuade the spirits rather than coerce them. The patient is usually associated for the rest of his life with the particular spirit or spirits that caused the illness. Because there are overtones of an alliance with evil powers, the patient has the responsibility to satisfy this zār or jinn with a special ritual at least once per year. 

According to Kennedy, the zār ritual in Nubia, which is mainly an adult female activity, reflects Nubian social conditions in terms of sex segregation, gender inequality, low female status, the restriction of women from religious participation, relative isolation, and marital insecurity. By observing the non-sick participants, he also supports his hypothesis that zār and other ritual activities allow not only the sick persons (possessed ones) but also the normal persons to be relieved of persistent and regular anxieties and tensions arising from Nubian life conditions. The common symptoms of the zār-possessed person include apathy, withdrawal from human company, minimal communication, refusal to work, strong desire to die, anorexia, and insomnia. Anxiety reactions or hysteria reactions like paralysis are also seen among the majority of the zār patients, and dissociative states are an outstanding characteristic of the zār ceremony. Although the zār ritual is specialized for healing these cases, a few psychotic patients are reported to have been healed as well. It is also assumed that the ceremony is like an acting-out of wish-fulfillment fantasies on the part of the women participating; the spirit who possessed the person demands material favours such as jewellery, new clothes, or expensive food, which the husband is expected to provide. The audience’s responses provide social support and temporarily indulge the possessed person’s wish fulfillment. Although ethnographers suggest that most patients are prone to hysteria or anxiety, the cult members feel that wass wassa (a depressive condition) is healed by zār (6, 36).

*Sudan:*

In Sudan, possession is usually associated with an illness that can be caused or intensified by spirits. However, it is not the case of every sickness. Doing ethnography on zār possession in the Hofriyati community of northern Sudan, Boddy states that the villagers attributed the illnesses to the variety of factors resulting from natural causes and that only the stubborn symptoms such as persistent headaches, nausea, anorexia, lassitude, apathy and depression, sleeplessness, anxiety, unspecified aches and pains, being easily saddened, and fertility problems may be ascribed to zayran (different kinds of zār). Similar to Kennedy’s observations in Nubia, some hysterical conversion disorders (as defined in the West) are reported in Hofriyati (e.g., blindness or paralysis of one or more limbs without diagnosed biological cause). These conditions are mainly associated with the zār possession. However, Hofriyati people might also link them to other supernatural causes, like sorcery (amal) or the evil eye (ayn harra), that make people more susceptible to spirit possession. In the Hofriyati, someone who is considered to be zār-affected is called either inda zār or inda dastur, which means “she has a spirit,” or is said to be mazur or madastir, which means “with a spirit,” possessed. Zār possession is known as an affliction and is expressed as an illness. Moreover, it is believed that zayran never abandon their hosts’ body, which means a person who is possessed will be always possessed and will be affected by her spirit(s) at any time. Villagers say if zayran infiltrate their hosts’ body, it will coincide with the latter's entrancement. The spirit's forceful entry into the body, which Hofriyatis called ghaybiya or ghaybuba (possession trance), displaces the person’s self to another perceptual stage. A trance is only one of many manifestations of possession as zayran affect their hosts in various ways (see also 4, 28). The possession trance usually occurs inside of the ritual contexts. In this situation, “One is not diagnosed as possessed because she becomes entranced; rather, she becomes entranced because she is possessed” (34, 35).

In Sudan, the possession ritual is not considered exclusive to women. However, spirits are attracted to married women, mostly between the ages of thirty-five and fifty-five, and rarely affect unwed women, whose fertility has not been activated yet. Spirits are also known to covet those women who use henna, perfumes, soaps, and scented oils, and wear gold jewelry and diaphanous wraps. As Boddy observed (1989), “[T]he proclivities of zayran are symmetrical to those of Hofriyati women: both are regarded as consumers of goods provided by men (p.141) (1).” Lewis (12, 15) claims women use zār possession as a strategy to cope with their subaltern social position. However, Boddy argues that this sexual disproportion among possessed persons is attributed to the link between the possession and fertility, which women are identified with and are responsible for (1).

***Zār in Iran***

Despite the variety of spirit possession rituals, people from southwestern to southeastern Iran believe in metaphysical forces. In these regions, people believe in and practice zār; however, there are varieties in the zār cults. In Baluchistan, in the southeast of Iran, these harmful winds are usually called Gowat (wind or air), which is equal to zār in the south (37). According to the people in these regions, most types of zār cause disease, discomfort, and serious chronic illnesses for the afflicted. They believe that the zārs could be contagious and people can give their zār to those whom they love or hate. Similar to the African zār cults, these people also believe in the eternality of zārs, which means that one who is possessed will be always possessed but can only come to terms with the spirits to leave the victim alone. In these cults, the zār ritual is not a female practice, and the poor and deprived people such as fishermen seem to be the most common victims (38, 39).

The origin of Iran’s Zār: The word “zār” in Farsi means desolate, feeble, languid, and weak; and it is derived from Persian rather Arabic (18) or more plausibly—given the long establishment of the cult in Gondar and vicinity—Amharic (6). However, according to Boddy (1989), the term zār probably originated from the Arabic zahar, meaning ‘he visited.’ In Hofriyat, it is often pronounced as zahr, meaning ‘he became visible, perceptible, or manifest.’ Both shifts are applicable in the possession context, where spirits become visible by entering the human realm via human bodies, temporarily displacing those bodies' human selves. Although there is a marked resemblance amongst the Zār rituals in Iran and many African countries, the origins of the zār cult and its name are obscure. 

These similarities suggest a common origin for this belief and practice (19). In the 20th century, scholars pointed to an Ethiopian or Abyssinian origin for zār; however, other origins, like Persian, have been also theorized (40, 41). Frobenius assumed common cultural traits between Persia, northeast Africa and the Sudan region and hypothesized that zār and bori were manifestations of an early system of beliefs originated in Persia (42), but because of the dominant presence of Africans amongst the cult members, it has been suggested that this belief and ritual might have been imported from Africa to the southwest of Iran (18, 40, 43). Studying the historical roots of the zār-bori cults, Lewis states, “[T]his cult or complex of cults, has its roots in Islamic West Africa (principally Nigeria and Niger), source of the bori component, and in Ethiopia, with its mixed Christian and Islamic heritage, source of zār. In Sudan, both these currents flow together forming, in the presence of local tumbura and other local cults, the hybrid zār-bori which has in turn spread to north Africa and Middle East”(15). Not all scholars agree with this hypothesis about the origin of Zār cults, and some believe that this spirit possession cult originated in Persia (39). It has been said that Persians applied the name zār to the cult when African sailors from the southeast coast of Africa introduced this ritual to them in the 16th century (18, 19). However, Mirzai Asl (2002) stresses that spirit-possession beliefs and practices such as zār, gowat, and liwa derived from Africa and spread wherever the enslaved settled. Based on this assumption, in the 19th century, as a result of the slave trade from Africa to Iran, the zār rituals have been kept in Iran as part of the heritage of African slaves, allowing them to reconstruct their identity.

Slavery and Persian Zārs: Africans were enslaved, transported, and sold through the slave trade network in the African interior. Most enslaved Africans were shipped through the sea trade route of the Indian Ocean, which ran from the Swahili coast to Muscat, the Ottoman Empire, the Arab States, and Iran. Thus, most of the Africans sent to Iran through the slave trade came from northern and eastern Africa through the Persian Gulf. Moreover, Iranian pilgrims brought another group of slaves from the Arabian cities of Baghdad, Karbala, Mecca and Medina to western and southwestern Iran. The landed slaves were taken to the customs houses at the Iranian ports and were sent from there to various cities of Iran, which had specialized slave markets in the 19th century. Local people in southern cities bought most of the enslaved Africans. A small number of slaves were also sent from southern ports to other parts of the country and were absorbed in different socioeconomic sectors. Most of the slaves who settled along the Persian Gulf coast worked as pearl fishers, laborers, or domestics. Some of them settled in the capital city and other big cities and were engaged in specific tasks in the harems of the Shah and princes. In harems, African male slaves served as lala (male nurses) and female slaves as daya (nanny) and dada (female nurses) and their children were called khanazad (house-born) (39).

The prophet Mohammad rejected the idea of slavery. The Quran established rules related to the treatment, sources, marriage, and emancipation of slaves. In Islam’s view, slaves have to be considered as a part of the household and had to be absorbed into the society after they converted to Islam. As converting non-Muslims to Islam and freeing the slaves are advocated and valued by Mohammad, Iranians had the religious justification to employ slaves, convert them to Islam, and then treat them as equals with the Iranian employees. This religious belief helped Persians to consciously justify enslavement; however, they might have treated the slaves as a part of their household. 

The trade of African slaves to Iran shaped the diaphonic communities of Afro-Iranians along the coast of the Persian Gulf in the southern parts of the country. However, because the slaves were integrated into a society dominated by non-blacks, they were invisible in certain areas of Iran. Nevertheless, the African heritage has been kept alive by the survival of the Afro-Iranian community in a few regions in southern Iran. Slavery and the diasporas of Africans from eastern and northern Africa allowed the slaves to introduce their cultural beliefs and practices to wherever they settled. These cultural features and traditions helped them preserve their heritage (39, 44). In southern Iran, the two societies of Africans and Iranians shared their cultures. As a result, the values and beliefs of enslaved Africans did not dominate the host culture but were integrated into the new culture. Despite the cross-cultural variations, the imported African culture underlined principles representing African unity and Africans’ supernatural beliefs. For example, the presence of different African healing cults distributed in different countries bordering the Indian Ocean including Iran exemplifies African cultural exportation (45). As shown earlier in this paper, zār has been practiced in Sudan, Egypt and Ethiopia, especially in the Gurage region and among the people of Gurage and Shoa. Iranian literature also attributed the ritual of the zār spirit possession in Iran to the enslaved Africans (41). However, since the mid-1920s, when the Iranian government (mainly Iran’s Ministry of Health) banned the practice of spirit possessions, most of these rituals have gradually become undetectable (18).

Zār Ritual in Iran: The zār ceremony has changed over time and from place to place; however, two common phases are recognized in most of the zār rituals along the Persian Gulf coastline: separation and incorporation. Sabaye Moghaddam in the Encyclopædia Iranica explains these phases (46). According to her, the separation phase that refers to the preparations for the zār begins with a person complaining about feelings of disease and discomfort to Bābā zār or Māmā zār; Zār cult leaders, who in African countries are called Sheikh (male leader) and Sheikha (female leader), in Iran are called Bābā zār or Māmā zār. All cult leaders are black, and some of them have already been possessed by zārs and have managed to control them. Most of the cult leaders believe that the patients should join the zār only when their doctors cannot treat them. However, there are some cult leaders who believe that the needles from the injections prescribed by the doctor will further agitate the zār and create more problems for the patient. When Bābā or Māmā zār chooses someone to heal, the patient will stay in the Bābā or Māmā zār’s home for up to seven days. During this separation phase, only Bābā zār or Māmā zār can visit the patient; moreover, the patient’s body is washed with seawater and every night Bābā zār or Māmā zār rubs a combination of aromatic herbs and spices on the patient’s body. In the last day of the separation phase, when the patient’s body is cleaned and washed to be prepared for the incorporation phase, one of the cult members, who is usually the one who has formerly been possessed by zār, informs everyone about the upcoming zār ceremony. Ahl-e-Hava (Eve’s family), who are people who have already been possessed by the zār and have been treated at least once, have to attend every zār ritual. In addition, other people of the cult, who may or may not be possessed, also participate as it is considered a sin not to attend a zār ritual. Depending on how big the Bābā or Māmā zār’s home is, the ritual can be inside or outside the house. Participants gather in a U-shaped open area—Maidān—with a patient sitting in the center. A piece of tablecloth including eggs, confetti, dates, and aromatic herbs is placed on the floor in the center of Maidān. After Bābā or Māmā zār covers the patient’s head with a piece of white cloth, a big tray holding aromatic herbs on burning charcoal is passed around and the patient and all participants are frequently incensed with the smoke from the mixture. The zār leader—Bābā or Māmā zār—leads the music, which involves local drums played by musicians and which is followed by others who are present at the ceremony. The leader is someone who knows the name of the zārs and its related music (the specific beat of drums). The leaders also sing and the participants respond to them. Songs of the incantations can be in a different language or just melodic sounds without lyrics. During the singing, when the zār hears its related music, it makes itself known through the body of the patient, who feels a strong inner urge to move. Every spirit has its own piece of music and some members of the cult, mainly Ahl-e-Hava, may start moving and shaking with every piece of the music, as they are supposed to be the eternal hosts of the zār. The leader cannot identify the zār until the patient reacts to the music, so musicians change the tune until the zār takes over the afflicted; the signs of this occurrence appear when the upper body and the head swing and the shoulders shake. 

When the zār is identified, Bābā or Māmā zār starts to communicate with the zār in a language that is ordinarily unfamiliar to themselves and the patient. This language is a combination of Persian, Arabic, Swahili, and Indian. Through this communication with the zār, the healer tries to find out the reasons behind the affliction and what the spirit wants in exchange for leaving the patient alone. Through the afflicted, the zār names its demands, which vary from simple things, such as a few prayers or a piece of bamboo (ḵeyzārān), to something more considerable, such as a sacrifice. Bābā/Māmā zār assures the zār that its demands will be met by tying a piece of cloth around the patient’s arm. It is believed that if the zār’s wishes are not granted, the zār will return and make more trouble for the patient. Depending on how easily the demand of the zār can be fulfilled, it will be quickly provided during the ceremony or will be met in a similar ritual later. For example, if the zār requests a sacrifice and that sacrifice can be provided quickly, then the cult members slaughter the animal; and after that, the patient, healer, and Ahl-e-Hava drink the blood. If the animal cannot be provided, then in another zār ceremony, this patient will ride in on an animal, which later will be sacrificed. The patient is then considered a cult member (Ahl-e-Hava) and must participate in all future ceremonies. These ceremonies can last for a week after the separation phase. There are also certain rules for the cult members: their clothing must be always clean and white and they must not touch the animal or human corpses, drink alcohol, or have sex outside their marriages. Also, they cannot sell or give away the object the zār has asked for, and if it is portable (e.g., clothing or an accessory), the patient must bring it to all future ceremonies. Otherwise, the zār will possess the patient again and the ceremony has to be repeated (also see Reading Saedi's AHL-E Hava by (47).

According to the people of southern Iran, the zār practice has changed over time from a simple ceremony that needed only a sheep to sacrifice to a more luxurious and expensive ritual. Moreover, since the mid-1920s, when the Iranian government, through reports provided by the Ministry of Health, forced the spirit believers to reduce the extent of the ceremonies, most of the zār cults disappeared from many regions, including Bushehr. The development of modern medicine and hospitals in Iran has also influenced the way people in these areas viewed and dealt with their health problems; however, zār beliefs and rituals have not completely disappeared from Persian Gulf islands like Queshm (44, 48).

## Conclusion

Zār (harmful “wind”) refers to the spirit possession that causes illness. Believing in the zār illness and the practice of the zār ritual as a therapy originated in Northern and Eastern Africa and was transported to other countries in the Middle East (such as Kuwait and Iran) through slavery. In most African countries, zār is mainly an adult female activity—involving married women mostly between the ages of thirty-five and fifty-five—which reflects the condition of sex-separation, low female social status, marital insecurity and relative isolation. Young states that the woman seeks remission from a sickness caused by the zār (9). She remains in a cult because she knows that when she is chosen by the zār, she will be vulnerable; and to control the disturbance, she has to participate in the cult activities. However, the psychic and social advantages that a woman gains from participating in the cult are related to the special characteristics attributed to the zārs. Similar to the African cults, in Arab countries like Kuwait, it has been reported that zār attracts middle-aged and middle-class women who have become isolated through the westernization of the society and who are looking for their familiar traditional world (15). This sexual disproportion could be due to political, economic, and social transformations in Hausaland that led to the social exclusion of some middle-aged Muslim women. Participation of this group of women in the bori cult is explained as their strategy to deal with a male-dominated world (39).

Nevertheless, in southern Iran, women are not the only ones who are affected by zār. In the regions where the zār cults still exist, everyone is subject to the action of the zār, but the poor and the deprived, such as fishermen, seem to be the zārs’ most common targets (18, 39). Although the prevalence of psychological disorders among the people of southern Iran has been attributed to the hardship in their lives (37), people with specific social status or gender participate in parallel spirit possession rituals in different corners of the world; this suggests that participating in such ceremonies has other reasons besides healing. The trance state of patients in the zār ceremony gives them a chance to shift the responsibility of disturbing behaviours and feelings from themselves to the spirits. Connecting to the zār cult also gives people the possibility to connect with the cult members who have suffered similar problems and allows them to observe their cultural values in a culturally safe atmosphere. 

Zār was considered as a culture-bound syndrome in DSM-IV; however, due to some innovations in DSM-V that led to a change in the notion of culture-bound syndromes and the appearance of cultural concepts of distress, the zār lost its place on the list of relevant examples. Several generations of anthropologists and cultural psychiatrists have tried to understand whether the languages that people use to explain their suffering can be brought into the psychiatric realm as conceptual tools. In the latest version of DSM, few disorders that are truly “bound” to one culture have been identified. Luis-Fernandez points out that these disorders are seen in different cultural contexts with the similar presentations, and though rarely, there are different cases across very diverse societies. He also emphasizes that all disorders, regardless of the context, are influenced by the cultural concept of the patient (49).

Balhara (2011), in his study on dhat syndrome—another formerly-called culture-bound syndrome and newly-called a cultural concept of distress—adds that even though these psychiatric disorders are classified under the same diagnostic category, various local cultural beliefs, attitudes, and knowledge generate different manifestations and illness models of these disorders in different cultures (50). According to him, one of the reasons behind the limited published literature on these conditions (e.g., no published randomized trial of interventions for dhat) is that they were categorized as culture-bound conditions, which led them to be considered as specific to certain regions. Moreover, categorizing such conditions, e.g., culture-bound syndromes, is unlikely to improve the management of these conditions (51). In contrast, cultural concepts offered by the Cross-Cultural Issues Subgroup (CCIS) of DSM-V bridge this gap by providing clinicians with the opportunity to obtain useful clinical information, to improve clinical rapport and engagement, and to improve therapeutic efficacy (49). Furthermore, cultural concepts guide clinical research that can facilitate the integration between cultural and clinical knowledge by identifying patterns of comorbidity and underlying biological substrates. In terms of cultural epidemiology, “distinguishing syndromes, idioms, and explanations provides an approach for studying the distribution of cultural features of illness across settings and regions, and over time. It also suggests questions about cultural determinants of risk, course, and outcome in clinical and community settings to enhance the evidence base of cultural research” [p.759] DSM-V(23).

Finally, due to the rapid increase in globalization and cultural diversity, particularly in Western societies, cases with any or all of the three new terms listed within the cultural concepts of distress—including culturally shaped or influenced symptom clusters; cultural idioms of distress; and explicit causal models, attributions, or explanations—are more likely to seek mental health care in different cultural contexts. Moreover, as it is mentioned in DSM-V’s cultural formulation chapter, cultural concepts provide clinicians with a framework that can help them to avoid misjudging the severity of a problem or assigning the wrong diagnosis because of its cultural variation in symptoms and/or in explanatory models (e.g., unfamiliar spiritual explanations such as zār may be misunderstood as psychosis). Given that clinical presentation of a patient can be influenced by cultural concepts and modes of expression, it is crucial for clinicians to be able to understand the sociocultural and historical contexts of where the illness is developed and how people in those contexts perceive their own mental health suffering.

## Funding

Mianji Fahimeh: Doctoral Award; Global Health Research-Capacity Strengthening Program (GHR-CAPS) – Trainee Award; McGill Institute for Health and Social Policy.
